# Kinetic, isotherm and thermodynamic studies on biosorption of chromium(VI) by using activated carbon from leaves of *Ficus nitida*

**DOI:** 10.1186/s13065-016-0180-1

**Published:** 2016-05-31

**Authors:** Ismat H. Ali, H. A. Alrafai

**Affiliations:** Department of Chemistry, College of Science, King Khalid University, P. O. Box 9004, Abha, 61321 Saudi Arabia; Department of Chemistry, College of Science for Girls, King Khalid University, P. O. Box 9004, Abha, 61321 Saudi Arabia

**Keywords:** Biosorption, Cr(VI), Isotherm, Kinetics, Thermodynamics, *Ficus nitida* leaves

## Abstract

**Background:**

Kinetics, thermodynamics and equilibrium of the removal of chromium(VI) ions from aqueous solutions by using chemically activated leaves of *Ficus nitida* were investigated. Adsorption runs were performed as a function of pH, mass of biosorbent, contact time, initial concentration of chromium(VI) ions and temperature.

**Results:**

The optimum conditions for maximum removal of chromium(VI) ion from aqueous solutions (about 99 %) were found to be 0.80 g of chemically activated leaves of *F. nitida*, 25 min, 50.0 mg/L of initial concentration of chromium(VI). Values of thermodynamic activation parameters proved that the biosorption process is spontaneous and endothermic. Results were analyzed by using Langmuir, Freundlich and Temkin models.

**Conclusions:**

Results of the study showed that the chemically activated leaves of *F. nitida* can be used as low cost, ecofriendly and effective sorbent for the removal of chromium(VI) from aqueous solutions.

**Graphical abstract:**
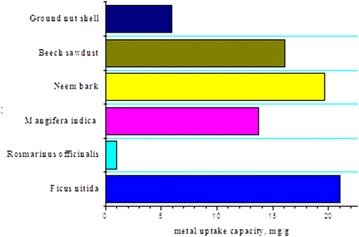
Ficus nitida is an efficient bio-sorbent used for removal of Cr(VI) ion

## Background

In the recent years the activities of industrial sectors has showed a considerable spread and development, but concurrently the natural environment has been contaminated. Heavy metals are one of the most widespread pollutants which contaminate the environment and cause serious damage to the ecosystem and also may be a reason for various dangerous diseases suffered by animals and human beings [1]. A number of industries are causing heavy metal pollution e.g. battery manufacturing processes, mining and metallurgical engineering, dyeing operations, electroplating, nuclear power plants, tanning, production of paints and pigments [2]. Heavy metals that may be considered as risky environmental pollutants are Cd, Hg, Pb, As, Cr, Hg, Ni and Cu. Comparing with organic pollutants, heavy metals are normally refractory and cannot be degraded or easily detoxified [3].

Chromium(VI) is one of the most poisonous contaminants which cause severe diseases and very harmful environmental complications. When chromium(VI) accumulates at high levels, it may lead to serious problems and even be fatal when concentrations reach 0.10 mg/g of body mass [4]. Chromium(VI) is more toxic than chromium(III) and as such receives more attention. Strong exposure to chromium(VI) has been linked to various types of cancer and may cause epigastric pain, nausea, vomiting, severe diarrhea and hemorrhage [5].

The removal of toxic metals from wastewater has been achieved using various methods like ion electro dialysis [6], sedimentation [7], ion exchange [8, 9], biological operations [10], coagulation/flocculation [11], nanofiltration technology [12], solid phase extraction [13], adsorption by chemical substances [14, 15] and electrokinetic remediation [16]. All these techniques suffer from multiple drawbacks such as high capital and operational costs and disposal of residual metal sludge [17]. In contrast, the bio-sorption method has become one of the most favored ways to remove heavy metals because it is environmentally friendly, highly efficient and has low associated costs. Various parts of plants are commonly used as biomass adsorbent for Cr(VI) adsorption from drinking water and wastewater. These include Syzygium jambolanum nut [18], Sophora japonica pods powder [19], rice bran [20], neem bark, neem leaves, rice straw and rice husk [21], gooseberry seeds [22], husk of Bengal gram [23], Cupressus lusitanica Bark [24] and Azadirachta indica [25].

Activated carbons are more effective in the removal of heavy metals ions because of some specific characteristics that augment the use of activated carbon for the removal of pollutants including heavy metals from water supplies and wastewater [17]. The ability of activated carbon to remove Cr(VI) by adsorption was reported many times. Activated carbon derived from procumbens [26], oil palm shell charcoal [27], groundnut hull [28], Sweet lime fruit skin and bagasse [29] were used for removal of Cr(VI) from aqueous solutions.

The aim of this study was to prepare activated carbons derived from leaves of *Ficus nitida* (AFNL) by chemical activation using H_2_SO_4_ and to use this activated carbon in removal of Cr(VI) ions from aqueous solutions.

## Experimental

### Preparation of biomass adsorbent

Leaves of *F. nitida* were collected from the main campus of King Khalid University, Abha, Saudi Arabia in September 2015. Leaves were thoroughly washed with distilled and deionized water, dried at room temperature for 3 days. The dried leaves were ground in an electric mill and then mixed with concentrated sulfuric acid in a mass ratio of 1:1.8 biomass:acid [17], then the mixture was filtrated and the obtained activated carbon was rinsed thoroughly with deionized water to remove the acid residue and dried for 6 h at 105 °C.

### Preparation of Cr(VI) solutions

Stock solution of potassium dichromate of 1000 mg/L concentration was prepared by dissolving the appropriate weight in 1.0 L of deionized water. The required concentrations were then prepared by taking adequate volumes from the stock solution.

### Batch bio-sorption study

Batch bio-sorption experiments were carried out by mixing bio-sorbent with Cr(VI) ion solutions of chosen concentration in 250 mL glass stoppered flask. A temperature controlled shaker at a speed of 120 rpm/min was used throughout all runs. The effect of pH on the adsorption of chromium(VI) ions was studied by using HCl and/or NaOH. The amount of bio-sorption was determined based on the difference between the preliminary and final concentrations in each flask as shown in Eq. (1)

1 $${\text{q}}_{\text{e}} = \, \left( {{\text{C}}_{\text{o}} {-}{\text{ C}}_{\text{e}} } \right){\text{V}}/{\text{M}}$$

where q_e_ is the metal uptake capacity (mg/g), V is the volume of the Cr(VI) solution in the flask (L) and M is the dry mass of bio-sorbent (g). Percent removal (% R) of Cr(VI) ions was determined by using of Eq. (2)

2 $$\% {\text{R }} = \, \left( {{\text{C}}_{\text{o}} {-}{\text{ C}}_{\text{e}} } \right) 100/{\text{V}}$$

### Instrumentation

pH measurements were carried out by using pH meter Hanna 211. Equilibrium concentrations were measured by using flame atomic absorption photometer (Spectra AA 20) in an air-acetylene flame. Chromium hollow cathode lamp was used as the radiation source with lamp current of 7 mA, wavelength of 357.9 nm and slit width of 0.2 nm. The specific surface area was measured using a SA-9601 analyzer.

### Reliability of results

A calibration curve was obtained using 0.5–4 mg/L concentration range of Cr(VI) ions. Linearity was calculated in order to investigate the reliability of results. Limit of detection LOD and limit of quantification LOQ were determined by reported method [30]. Precision was verified by determination of relative standard deviation RSD and accuracy was checked by recovery study.

## Results and discussion

### Reliability of results

A number of parameters i.e., linearity, LOD, LOQ, RSD were determined in order to check the reliability of results.

### Linearity

The linearity of the calibration curve was evaluated by plotting the absorbance of standard solutions of Cr(VI) against the concentration. A straight line with regression coefficient (R^2^) of 0.997 was obtained indicating good linearity.

### LOD and LOQ

Sensitivity was evaluated by determination of limit of detection (LOD) and limit of quantitation (LOQ). (LOD) and (LOQ), were determined by measuring 10 blank samples. By using the relationships 3.3SD/b and 10SD/b, it was found that LOD = 0.02 mg/L and LOQ = 0.06 mg/L, respectively.

### Precision

The relative standard deviation (RSD) usually expresses precision of measurements. Practically, precision is determined by evaluating the reproducibility of the results. Ten blank samples were measured at the same conditions and the obtained RSD value was 7.05 % which is in the acceptable limit [31].

### Accuracy

Usually recovery studies are carried out in order to check the accuracy. Recovery studies were performed by spiking technique. The recovery value, determined as 93.2 %, is within the acceptable range [32].

### Surface area of AFNL

The BET surface area analysis revealed that AFNL has a specific surface area of 1230 m^2^ g^−1^ indicating that AFNL may have good metal uptake capacity.

### Effect of pH

**Fig. 1:**
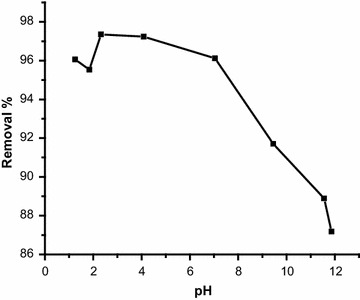
Influence of pH on the removal of Cr(VI) ions

The pH of the solution is one of the factors that may affect bio-sorption of heavy metals. Figure 1 shows that bio-sorption of Cr(VI) onto ALFN is dependent on the pH of the solution. Maximum removal of Cr(VI) ions from aqueous solution was achieved at acidic pH range. The optimal pH range for Cr(VI) removal was from 1.50 to 4.00. When the pH value is greater than 6.00 it is likely that Cr(VI) ions were precipitated as a result of the formation of hydroxides and thus removal efficiency decreased sharply. At lower pH values, protons exist in high concentration and binding sites of metals became positively charged and this has a repelling effect on the Cr(VI) cations. As the pH value increases, the density of negative charge on AFNL rises because of deprotonation of the binding sites in the metals, hence increasing metal uptake. This is in good agreement with the previous explanations [17].

### Effect of biomass weight

**Fig. 2:**
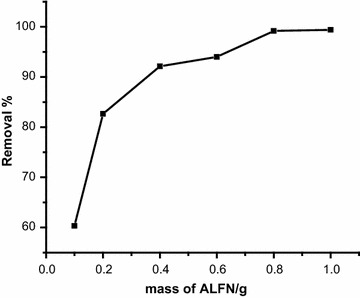
Effect of amount of ALFN on the removal of Cr(VI) ions

The bio-sorbent quantity is a significant factor because it may control the metal uptake capacity of a bio-sorbent for a given concentration. The bio-sorption effectiveness for Cr(VI) ions as a function of bio-sorbent amount was examined. A number of solutions were prepared with the adsorbent dose of 0.10, 0.20, 0.40, 0.60, 0.80 and 1.00 g/100 mL of chromium(VI) solution (50 mg/L). Figure 2 shows that the percentage of the metal bio-sorption clearly increases with the bio-sorbent mass up to 0.80 g/100 mL. Therefore, the optimum bio-sorbent dosage was taken as 0.80 g/100 mL for further experiments. This result can be attributed to the fact that the bio-sorption sites remain unsaturated for the period of the bio-sorption process, whereas the number of sites available for bio-sorption site increases by increasing the bio-sorbent dose. Furthermore when the bio-sorbent ratio is small, the active sites available for binding metal ions on the surface of *F. nitida* are less, so the bio-sorption effectiveness is low. As the bio-sorbent quantity increased, more active sites to bind Cr(VI) ions are available, thus it results an increase in the bio-sorption efficiency until saturation.

### Effect of contact time

The impact of contact time on the removal of 50 mg/L of Cr(VI) ions from aqueous solutions was also investigated. Results revealed that the metal ions removal increases linearly with time up to 25 min and then remains at the same level. The rate of metal ion removal is higher in the beginning because of the large surface area of the adsorbent available for the adsorption of the Cr(VI). Furthermore, no major changes were observed in the removal of Cr(VI) ions from the aqueous solution after 24 h of equilibration.

### Kinetic calculations

**Fig. 3:**
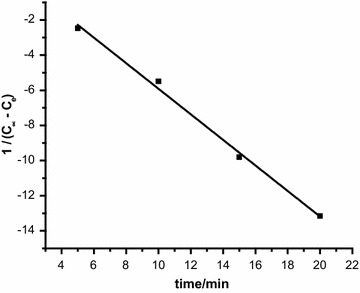
Pseudo-second-order kinetics for Cr(VI) ions onto ALFN

Kinetics of bio-sorption of Cr(VI) ions onto activated carbon of leaves of *F. nitida* was studied. It is obvious from the results (Fig. 3) that the bio-sorption behavior follows Eq. 3 indicating second order kinetics.

3 $$1/\left( {{\text{C}}_{\infty } {-}{\text{ C}}_{\text{e}} } \right) \, = {\text{ kt }} + { 1}/{\text{C}}_{\text{o}}$$

### Effect of interfering ions

An aqueous solution containing 50 mg/L of Cr(VI) ions, 5 mg/L of Pb(II) ions, 5 mg/L of Cd(II) ions and 5 mg/L of Ni(II) ions was used to study the effect of interfering ions on the efficiency of AFNL on removal of Cr(VI) ions. Results showed that after 30 min of shaking time, 96 % of Cr(VI) ions were removed from the aqueous solution indicating that the interfering ions have almost no effect on the efficiency of AFNL to remove Cr(VI) ions. Furthermore very small quantities of the interfering ions were removed demonstrating that AFNL may be used as selective bio-sorbent for Cr(VI) ions. This may be attributed to the fact that the experiment was carried out at the optimal conditions for Cr(VI) removal.

### Effect of Cr(VI) concentration

The effect of initial concentrations of Cr(VI) ions on its adsorption on the ALFN was investigated by varying the initial concentration from 50 to 200 mg/L. Results revealed that the removal percentage is inversely proportional to the initial Cr(VI) concentration. This may be attributed to coverage of active sites of adsorbent as the concentration of Cr(VI) increases.

Adsorption of Cr(VI) ions onto ALFN was studied using three models of adsorption isotherm: Langmuir, Freundlich and Temkin isotherms. The aim of adsorption isotherms is to explain the relation between the remaining concentration of the adsorbate and the adsorbed quantity on the sorbent surface.

### Langmuir isotherm

The Langmuir isotherm postulates monolayer adsorption on a uniform surface with a limited number of adsorption sites. Once a site is filled, no additional sorption can occur at that site [33]. The linear equation of the Langmuir isotherm model is described by Eq. (4).

4 $$\frac{q_e}{c_e} = \frac{1}{q_m\,b} + \frac{c_e}{q_m}$$

where q_m_ is the maximum adsorption capacity (mg/g) and b is the Langmuir constant which related to adsorption rate. Values of q_m_ and b are shown in Table 1. The attraction between sorbent and sorbate can be deduced by using separation factor, b, as shown in Eq. [5]:

5 $${\text{R}}_{\text{L}} = \frac{1}{1 + b\, Co}$$

R_L_ value provides significant evidence about the adsorption nature. Langmuir isotherm is considered to be irreversible when R_L_ is equal to zero, favorable when 0 < R_L_ < 1, linear when R_L_ = 1 or unfavorable when R_L_ > 1. R_L_ values were determined as 0.10, 0.07, 0.05, 0.04, 0.03 and 0.02 for concentrations 50, 70, 100, 120, 150 and 200 mg/L of Cr(VI) ions indicating favorable adsorption.

**Table 1:** Constants of different adsorption isotherm models

Isotherm	Value
Langmuir	
q_m_, mg/g	21.0
b, L/g	0.185
R^2^	0.9995
Frenudlich	
n	2.85
Kf, mg/g (L/mg)^1/n^	4.79
R^2^	0.9343
Temkin	
A, L/g	2.93
B	3.46
R^2^	0.9901

### Freundlich isotherm

This model is applied to adsorption on heterogeneous surfaces with the interaction between adsorbed molecules. Application of the Freundlich equation suggests that adsorption energy exponentially decreases on completion of the adsorption centers of sorbent. This isotherm is an empirical equation and can be employed to describe heterogeneous systems as shown in Eq. (6).

6 $${\text{ln q}}_{\text{e}} = {\text{ ln K}}_{\text{f}} + { 1}/{\text{n ln C}}_{\text{e}}$$

where K_f_ is the adsorption capacity of sorbent, n value determines the degree of non-linearity between solution concentration and adsorption in this manner: if n = 1, then adsorption is linear; if n > 1, then adsorption is a chemical process; if n < 1, then adsorption is a physical process. K_f_ and n values were listed in Table 1. The n value lies between one and ten indicating the physical adsorption of Cr(VI) onto ALFN.

### Temkin isotherm

Temkin isotherm [34] takes into consideration the indirect interaction between adsorbate molecules and assumes that the heat of adsorption of all molecules in the layer decreases linearly with coverage due to adsorbent–adsorbate interactions and that the adsorption is characterized by a uniform distribution of the binding energies up to a maximum binding energy. The Temkin isotherm model has been used in the linear form as shown in Eq. (7).

7 $${\text{q}}_{\text{e}} = {\text{ B lnA }} + {\text{ B ln C}}_{\text{e}}$$

where B = RT/b, b is the Temkin constant associated to heat of adsorption (J/mol), A is the Temkin isotherm constant (L/g), R is the universal gas constant (8.314) J/mol. K, and T is the absolute temperature (K). The constants B and A are listed in Table 1.

### Temperature effect

The effect of temperature on bio-sorption of Cr(VI) on ALFN was studied at temperature range of 25.0–50.0 °C. Equations (8–12) were used to calculate some thermodynamic parameters

8 $$\Delta {\text{G}}^{\text{o}} = \, - {\text{RT lnK}}_{\text{D}},$$

K_D_ is defined as:

9 $${\text{K}}_{\text{D}} = {\text{ C}}_{\text{o}} /{\text{C}}_{\text{e}}$$

10 $$\Delta {\text{G}}^{\text{o}} = \, \Delta {\text{H}}^{\text{o}} {-}{\text{ T }}\Delta {\text{S}}^{\text{o}}$$

Equations (8) and (10) can be written as:

11 $$- {\text{RT lnK}}_{\text{D}} = \, \Delta {\text{H}}^{\text{o}} {-}{\text{ T }}\Delta {\text{S}}^{\text{o}}$$

on rearrangement

12 $${\text{lnK}}_{\text{D}} = \, \frac{{-\,\Delta {\text{H}}^{\text{o}} }}{\text{RT}} \frac{{+\,\Delta {\text{S}}^{\text{o}} }}{\text{R}}$$

Enthalpy and entropy change of activation were calculated from Eq. (12), while values of free energy change of activation ΔG^o^ were determined from Eq. (8).

Table 2 showed that (ΔG^o^) has negative values indicating that the bio-sorption process is spontaneous. It is also observed that the negative values of free energy change, increases with increasing temperature. This may be ascribed to activation of more sites on the surface of ALFN with a rise in temperature or that the energy of bio-sorption sites has an exponential distribution band at higher temperature enabling the energy barrier of bio-sorption to be overcome. When the free energy change (ΔG^o^) ranges between −20 and 0 kJ/mol, adsorption is classified as physical adsorption, while in chemical adsorption values of free energy change range from −80 to −400 kJ/mol. ΔG^o^ for Cr(VI) bio-sorption onto ALFN was in the range of (−5.02 to −13.52) kJ/mol and so the adsorption was predominantly physical bio-sorption. This is in agreement with results derived from the n value calculated with the Freundlich isotherm. Results showed that the value of ΔS^o^ is 343.72 J/mol K. This positive value showed that there is an increased randomness at the solid solution interface during the adsorption of Cr(VI) ions onto ALFN. Results in Table 1 also showed that the bio-sorption is an endothermic process.

**Table 2:** Thermodynamic parameters of the biosorption of Cr(VI) ions onto ALFN

T, K	K_d_	ΔG^o^ (kJ/mol)	ΔH^o^ (kJ/mol)	ΔS^o^ (J/mol K)
298	153.61	−13.52	97.24	343.72
303	61.99	−10.74		
313	15.67	−0 6.93		
323	7.59	−0 5.02		

### Comparison of ALFN with other sorbents

Comparison of maximum biosorption capacity, q_m_ of ALFN with those of some other biosorbents stated in the literature is given in Table 3. Variances in q_m_ could be ascribed to the properties and nature of each biosorbent such as structure and surface area of the biosorbent. A comparison with other adsorbents proves that ALFN may be considered as a good biosorbent.

**Table 3:** Comparison of maximum uptake capacity for various bio-sorbents

Biosorbent	Metal uptake capacity, mg/g	Ref.
Activated carbon from *Ficus nitida* leaves	21.0	This study
Activated carbon from *Rosmarinus officinalis* leaves	1.0	[34]
*Mangifera indica* bark	13.7	[35]
*Syzygium cumini* bark	25.4	[35]
Neem bark	19.6	[36]
Beech sawdust	16.1	[37]
Walnut hull	98.1	[38]
Ground nut shell	5.9	[39]
Rice husk	0.6	[40]

## Conclusions

Biosorption of Cr(VI) ions onto activated carbon prepared from leaves of *F. nitida* was investigated and found to be dependent on pH value of solution, adsorbent mass, contact time, temperature and initial Cr(VI) concentration.

Data of biosorption of Cr(VI) on ALFN were applied to three adsorption isotherm models. The maximum adsorption capacity was determined from the Langmuir isotherm as 21.0 mg/g. The n value obtained from the Freundlich isotherm indicates that the sorption of Cr(VI) ions onto ALFN is favorable. Adsorption process of Cr(VI) ions onto ALFN was found to obey the second-order kinetic equation. Thermodynamic parameters proved that the adsorption process is spontaneous and endothermic.
